# Sleep disturbances are associated with specific sensory sensitivities in children with autism

**DOI:** 10.1186/s13229-018-0206-8

**Published:** 2018-03-27

**Authors:** Orna Tzischinsky, Gal Meiri, Liora Manelis, Asif Bar-Sinai, Hagit Flusser, Analya Michaelovski, Orit Zivan, Michal Ilan, Michal Faroy, Idan Menashe, Ilan Dinstein

**Affiliations:** 1 Behavioral Science Department, Emek Yesreel College, Emek Yesreel, Israel; 2 Pre-School Psychiatry Unit, Soroka University Medical Center, Beer Sheva, Israel; 3 Psychology Department, Ben Gurion University, Beer Sheva, Israel; 4 Zusman Child Development Center, Soroka University Medical Center, Beer Sheva, Israel; 5 Public Health Department, Ben Gurion University, Beer Sheva, Israel; 6 Cognitive and Brain Sciences Department, Ben Gurion University, Beer Sheva, Israel

**Keywords:** Autism, Children, Sensory abnormalities, Sleep disturbances, Hypersensitivity towards touch

## Abstract

**Background:**

Sensory abnormalities and sleep disturbances are highly prevalent in children with autism, but the potential relationship between these two domains has rarely been explored. Understanding such relationships is important for identifying children with autism who exhibit more homogeneous symptoms.

**Methods:**

Here, we examined this relationship using the Caregiver Sensory Profile and the Children’s Sleep Habits Questionnaire, which were completed by parents of 69 children with autism and 62 age-matched controls.

**Results:**

In line with previous studies, children with autism exhibited more severe sensory abnormalities and sleep disturbances than age-matched controls. The sleep disturbance scores were moderately associated with touch and oral sensitivities in the autism group and with touch and vestibular sensitivities in the control group. Hypersensitivity towards touch, in particular, exhibited the strongest relationship with sleep disturbances in the autism group and single-handedly explained 24% of the variance in total sleep disturbance scores. In contrast, sensitivity in other sensory domains such as vision and audition was not associated with sleep quality in either group.

**Conclusions:**

While it is often assumed that sensitivities in all sensory domains are similarly associated with sleep problems, our results suggest that hypersensitivity towards touch exhibits the strongest relationship with sleep disturbances when examining children autism. We speculate that hypersensitivity towards touch interferes with sleep onset and maintenance in a considerable number of children with autism who exhibit severe sleep disturbances. This may indicate the existence of a specific sleep disturbance mechanism that is associated with sensitivity to touch, which may be important to consider in future scientific and clinical studies.

## Background

Autism is a remarkably heterogeneous disorder where different individuals exhibit distinct behavioral symptoms. This heterogeneity is apparent in the core symptoms that define the disorder (i.e., impaired social communication/interaction, repetitive behaviors, restricted interests, and sensory abnormalities) [1] and in additional symptoms that are prevalent in individuals with autism (e.g., sleep disturbances). A major goal of contemporary autism research is to identify individuals who share more homogeneous symptoms and who may benefit from targeted interventions [2, 3]. Understanding potential relationships across symptom domains is an important step in characterizing individuals with more homogenous symptoms.

A large body of literature has shown that sensory problems are apparent in 60–90% of individuals with autism [4–9]. This has motivated the addition of sensory problems as a diagnostic criterion of autism in the DSM-5 [1]. However, sensory problems in autism can vary widely and include both hypo- and hypersensitivities in multiple sensory modalities [4, 6, 10–14]. Indeed, both hypo- and hypersensitivity can appear within the same individuals with autism at different times and in different contexts/situations [15]. Previous studies have shown that sensory abnormalities are positively correlated with autism severity in adults [16] and may [7] or may not [8] be correlated with adaptive behaviors in children with autism.

Sleep disturbances are another common symptom that is apparent in 40–80% of individuals with autism [17–20]. Disturbances include difficulty falling asleep, frequent wakings during the night, shorter sleep duration, and restlessness during sleep. Previous studies have reported that sleep disturbances are more prevalent in regressive autism [19], increase with autism severity [18, 21], and may [22, 23] or may not [17, 20] be associated with cognitive levels. In addition, the severity of sleep disturbances in children with autism seems to scale with their level of anxiety, attention deficits, impulsivity, challenging behaviors, and the use of medication [18, 21, 24–26].

Several studies have hypothesized that sleep disturbances may be associated with or even caused by sensory sensitivities in autism [19, 27], but this potential relationship has rarely been examined empirically. With this in mind, two recent studies used Autism Speaks’ Autism Treatment Network (ATN) to examine the potential relationship between sensory abnormalities and sleep disturbances in autism. The ATN is a large national database [28] containing a wide variety of behavioral information from children with autism, which includes the Child Behavior Checklist (CBCL) [29], Short Sensory Profile [30], and a short, 23 item version, of the Children’s Sleep Habits Questionnaire (CSHQ) [31].

Both studies reported that sleep disturbances were significantly associated with children’s anxiety levels as measured by the CBCL. In addition, one study reported that adding the under-responsive/sensory-seeking and auditory filtering scores of the Short Sensory Profile to a hierarchical regression model significantly improved the ability to predict total sleep disturbance scores from the CSHQ by 1% [21]. The second study computed a sensory over-responsivity score for each child (equivalent to sensory hypersensitivity), by summing the scores of several questions in the Short Sensory Profile that pertain to sensory hypersensitivity in multiple domains (i.e., touch, vision, taste/smell, and audition). This study reported significant correlations between sensory over-responsivity and several CSHQ subscales, which explained 1–6% of the variance in CSHQ scores [25]. Taken together, these studies suggest that Short Sensory Profile scores offer significant, but limited utility in explaining sleep disturbance scores of individuals with autism.

The goal of the current study was to perform a more in-depth examination of the relationship between sleep disturbances and sensory sensitivities by using the complete Caregiver Sensory Profile [32]. Unlike the Short Sensory Profile, which integrates scores across multiple sensory domains [30], the complete Caregiver Sensory Profile contains a larger number of questions that allow one to compute separate hypo- and hypersensitivity scores for each of five sensory domains (audition, vision, taste/smell, vestibular, and touch). This allowed us to determine whether sleep disturbances are more strongly associated with some sensory sensitivities than others. Determining such specificity has value for elucidating the physiological mechanisms that may generate sleep disturbances in autism and for guiding future clinical trials with sensory therapies and aids.

## Methods

### Participants

A total of 131 children participated in the study (Table 1): 69 children with autism (age 3–7, mean age 4.94 ± 1.23, 56 male) and 62 age-matched controls (age 3–7, mean age 4.82 ± 1.15, 41 males). There was no significant difference in the age of participating children across groups (*t*(129)) = 0.64, *p* = 0.57, two-tailed *t* test). Children with autism were recruited through the Negev Autism Center [33]. Control children of the same age were recruited from the community through an online forum at Ben Gurion University. Parents of all control children reported that their children were never suspected of having any developmental problems. Both the Helsinki committee at Soroka Medical Center and ethics committee at Ben Gurion University approved this study, and parents of all participating children signed an informed consent form.

**Table 1:** Sample characteristics

	Autism *n* = 69	Typically developing *n* = 62
Gender	56 males, 13 females	41 males, 21 females
Age (years)	4.94 (1.23)	4.82 (1.15)
ADOS social*	12.4 (5.2)	
ADOS repetitive behaviors*	4.02 (1.8)	
ADOS total*	16.4 (6.5)	
ADOS comparison score*	6.9 (1.92)	

Gender and age of autism and control children as well as ADOS scores from the 49 children with autism who completed the assessment

*n = 49

### Diagnosis

All children with autism met the DSM-5 criteria for autism as determined by both a physician (child psychiatrist or neurologist) and a developmental psychologist. Forty-nine of the 69 children with autism also completed an Autism Diagnostic Observation Schedule (ADOS) assessment to confirm the diagnosis [34]. Twenty-two of the 69 children with autism were taking medications that included Melatonin, Risperdal, Ritalin, and Neuleptil.

### Sensory profile

We used the Hebrew version of the Caregiver Sensory Profile questionnaire to assess sensory sensitivities in all children [35]. This questionnaire contains 125 questions that quantify the frequency of abnormal behavioral responses to various sensory experiences [32]. In the current study, we focused only on the five sensory subscales of the Sensory Profile, which include questions about auditory, visual, vestibular, touch, and oral sensory processing. These questions are split into high-threshold and low-threshold items, which measure hypo- and hypersensitivities respectively, with lower scores indicating more severe symptoms. We examined differences across groups for each of these scores separately and also for their total raw score, which combines both low- and high-threshold items, while keeping in mind that some children exhibit both hypo- and hypersensitivity to different sensory experiences (Tables 2 and 3).

**Table 2:** Sensory Profile scores

Sensory Profile					
	Autism	Control	*T* stat	*p* value	Cohen’s *d*
Auditory	24.30 (0.76)	33.4 (0.6)	*t*(129) = −9.37	*p < 0.001*	2.02
Visual	33.97 (0.78)	39.58 (0.57)	*t*(129) = − 5.78	*p < 0.001*	1.00
Vestibular	42.54 (0.75)	51.34 (0.47)	*t*(129) = − 9.97	*p < 0.001*	1.72
Touch	64.48 (1.54)	81.81 (0.82)	*t*(129) = − 9.95	*p < 0.001*	1.71
Oral	40.29 (1.28)	52.84 (0.91)	*t*(129) = − 7.97	*p < 0.001*	1.39

The mean and standard error (in parentheses) are presented for the autism (left column) and control (second column) groups along with the statistics of two-sample *t* tests with unequal variances (right hand column). Italicized *p* values were significant after Bonferroni correction (*p* < 0.01)

**Table 3:** Correlations across sensory domains

	Visual	Vestibular	Touch	Oral
Autism (*n* = 69)				
Auditory score	*P* = 0.5*	*P* = 0.46*	*P* = 0.46*	*P* = 0.44*
	*S* = 0.49*	*S* = 0.41*	*S* = 0.45*	*S* = 0.44*
Visual score		*P* = 0.47*	*P* = 0.46*	*P* = 0.5*
		*S* = 0.45*	*S* = 0.49*	*S* = 0.52*
Vestibular score			*P* = 0.67*	*P* = 0.52*
			*S* = 0.68*	*S* = 0.53*
Touch score				*P* = 0.63*
				*S* = 0.64*
Control (*n* = 62)				
Auditory score	*P* = 0.6*	*P* = 0.61*	*P* = 0.57*	*P* = 0.32
	*S* = 0.59*	*S* = 0.63*	*S* = 0.61*	*S* = 0.38*
Visual score		*P* = 0.53*	*P* = 0.55*	*P* = 0.38*
		*S* = 0.57*	*S* = 0.63*	*S* = 0.55*
Vestibular score			*P* = 0.75*	*P* = 0.51*
			*S* = 0.71*	*S* = 0.53*
Touch score				*P* = 0.47*
				*S* = 0.50*

Pearson’s (*P*) and Spearman’s (*S*) correlation coefficients are presented for each pair of sensory domains in the Sensory Profile for the autism (top) and control (bottom) groups. Asterisks indicate significant correlation coefficients after Bonferroni correction (*p* < 0.005)

### CSHQ

Parents of all participants scored their child’s sleep behaviors using the Hebrew version of the CSHQ [31, 36]. This caregiver questionnaire includes 33 items which are divided into eight subscales representing different sleep disturbances: bedtime resistance, sleep anxiety, sleep onset delay, sleep duration, night wakings, daytime sleepiness, sleep-disordered breathing, and parasomnias. The scores of these eight domains are summed to generate a total score of sleep disturbances for each child. The internal consistency values of the CSHQ subscales in our study were *α* = 0.533–0.758, and their consistency with the total sleep score was *α* = 0.838.

### Statistical analyses

All statistical analyses were performed with Matlab (Mathworks, USA). We performed two-tailed *t* tests with unequal variance to compare the five sensory measures (visual, auditory, vestibular, touch, and oral) of the Sensory Profile across the autism and control groups. Equivalent tests were performed for the sleep measures (bedtime resistance, sleep onset delay, sleep duration, sleep anxiety, night wakings, parasomnias, sleep-disordered breathing, daytime sleepiness, and total sleep score). All tests were corrected for multiple comparisons using the Bonferroni method (i.e., five subscales of the Sensory Profile and eight subscales in the CSHQ). All sensory and sleep measures were close to normal distributions as demonstrated by their skewness and kurtosis values, which were all between − 1 and 1, except for the visual and vestibular scores in the control group (skewness = − 1.48 and − 1.23, kurtosis = 4.38 and 1.59, respectively). Potential relationships between scores from each of the sensory modalities and the total sleep score were examined using both Pearson’s and Spearman’s correlations. This ensured that our conclusions were not based on the assumption that the distributions of the variables were normal or that the relationships were linear.

In a final set of analyses, we performed several regression analyses, separately for children with autism and controls, and separately for the total raw scores, low-threshold items, and high-threshold items of the Sensory Profile. In each case, we examined the ability of the sensory profile scores to explain the total sleep disturbance scores. Initial regression models contained a single predictor containing the scores from each of the five sensory domains (i.e., visual, auditory, vestibular, touch, or oral), and in a sixth model we included all five predictors together. This enabled us to examine the contribution of scores from each sensory modality, when attempting to explain the severity of sleep disturbances in individual children. In a final regression analysis, we also added age, gender, medication-use, and ADOS scores to determine whether these variables had an effect on the variance explained.

## Results

### Sensory sensitivities in autism

**Fig. 1:**
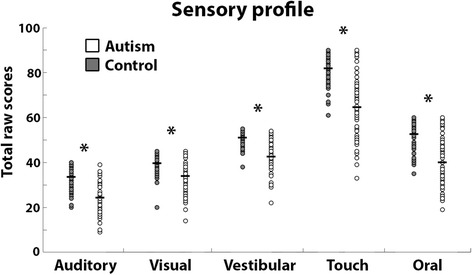
Scatter plots of sensory profile scores. The total raw scores from each sensory modality in the Sensory Profile are presented for children with autism (white) and controls (gray). Each circle represents a single child. Black lines indicate group mean. Asterisks indicate significant differences across groups after Bonferroni correction (*p* < 0.01)

Children with autism exhibited abnormal sensory sensitivities that were evident in the Sensory Profile scores of all five sensory modalities (Fig. 1 and Table 2). The total raw scores from the Sensory Profile were significantly lower in children with autism as compared to the control children in the auditory, visual, vestibular, touch, and oral domains (Table 2). Similar results were also evident in all five sensory domains when comparing only male children in the two groups (*t*(95) < − 4.7, *p* < 0.001), when including only children with autism who completed an ADOS assessment (*t*(109) < − 4.1, *p* < 0.001), or when excluding children with autism who were taking medications (*t*(107) < − 5.5, *p* < 0.001). Furthermore, sensory sensitivity scores from all five sensory modalities were not significantly different between children with autism who were or were not taking medication (*t*(41) < 0.9, *p* > 0.34), nor between male and female children with autism (*t*(20) > − 1, *p* > 0.29).

**Fig. 2:**
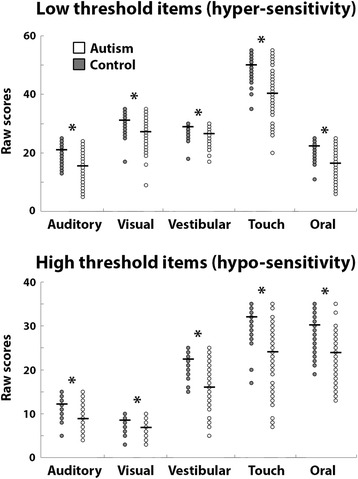
Hypo- and hypersensitivities. Scatter plots of low- (top) and high (bottom)-threshold item scores from the Sensory Profile for children with autism (white) and control (gray) groups. Each circle represents a single child. Black lines indicate group mean. Asterisks indicate significant differences across groups after Bonferroni correction (*p* < 0.01)

The total raw scores of the Sensory Profile are the sum of scores from both low- and high-threshold items on the questionnaire, which measure hyper and hyposensitivities respectively (Fig. 2). Note that children can be hypersensitive to some stimuli and hyposensitive to other stimuli even within the same sensory domain. Children with autism exhibited significantly lower scores on the low-threshold (hypersensitivity) items as compared with controls in all five sensory modalities (*t*(129) < − 4.55, *p* < 0.005). The same was also true for high-threshold items (*t*(129) < − 5.75, *p* < 0.005).

Equivalent results were found when including only male children, for both low- (*t*(95) < − 4.1, *p* < 0.001) and high-threshold items (*t*(95) < − 4.6, *p* < 0.005), when including only children who had ADOS scores, for both low- (*t*(109) < − 3.65, *p* < 0.01) and high-threshold items (*t*(109) < − 4.5, *p* < 0.001), and when excluding children with autism who were taking medication, for both low- (*t*(107) < − 4.8, *p* < 0.001) and high-threshold items (*t*(107) < − 5.2, *p* < 0.001).

Strong and significant correlations were found between Sensory Profile scores of different sensory domains in both the autism and control groups (Table 3), demonstrating that sensory sensitivities of individual children were similar across most sensory domains in both groups.

### Sleep problems in autism

**Fig. 3:**
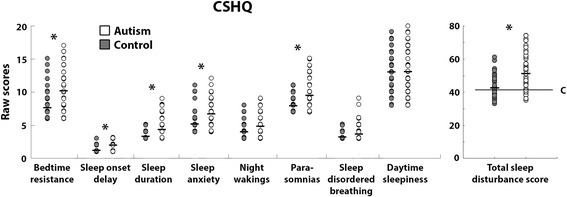
Scatter plots of CSHQ scores. Specific subscales (left) and total sleep disturbances (right) are presented for children with autism (white) and controls (gray). Each circle represents a single child. Black lines indicate group mean. Asterisks indicate significant differences across groups after Bonferroni correction (*p* < 0.006). C indicates the cutoff line that is often used when screening children for clinical sleep problems [27]

Children with autism exhibited significantly larger sleep disturbance scores than control children in the total score and in all CSHQ subscales, except for sleep-disordered breathing, night wakings, and day time sleepiness (Table 4 and Fig. 3). A total sleep disturbances score of 41 is considered to be a useful clinical cutoff when screening children for sleep problems [27]. Significantly more children with autism (85.5%) had scores higher than this cutoff in comparison to control children (54.8%) (*X*^2^(1) = 14.91, *p* < 0.001).

**Table 4:** CSHQ scores

CSHQ scores					
	Autism	Control	*T* stats	*p* value	Cohen’s *d*
Bedtimes resistance	10.26 (0.39)	7.66 (0.29)	*t*(129) = 5.35	*p < 0.001*	0.93
Sleep onset delay	1.97 (0.10)	1.26 (0 .06)	*t*(129) = 6.03	*p < 0.001*	1.04
Sleep duration	4.38 (0.20)	3.31 (0.08)	*t*(129) = 4.88	*p < 0.005*	0.84
Sleep anxiety	6.73 (0.25)	5.24 (0.21)	*t*(129) = 4.54	*p < 0.005*	0.79
Parasomnia	9.49 (0.27)	7.92 (0.15)	*t*(129) = 5.13	*p < 0.001*	0.88
Night wakings	4.86 (0.20)	4.0 (0.16)	*t*(129) = 3.27	*p* < 0.01	0.57
Sleep-disordered breathings	3.64 (0.15)	3.23 (0.07)	*t*(129) = 2.48	*p* = 0.12	0.43
Day time sleepiness	13.1 (0.40)	13.05 (0.34)	*t*(129) = 0.1	*p* = 0.92	0.02
Total sleep disturbances	50.74 (1.13)	42.86 (0.73)	*t*(129) = 5.87	*p < 0.001*	1.02

The mean and standard error (in parentheses) are presented for the autism (left column) and control (middle column) groups along with the statistics of a two-sample *t* tests with unequal variances (right hand columns). Italicized *p* values were significant after Bonferroni correction (*p* < 0.006)

Performing the same analysis while including different subsets of children with autism yielded similar results. Total sleep disturbance scores were significantly larger in the autism group when excluding children with autism who were taking medication (*t*(107) = 3.23, *p* = 0.001), when including only male children in both groups (*t*(95) = 5.09, *p* < 0.001), and when including only children who completed the ADOS in the autism group (*t*(109) = 4.3, *p* < 0.001).

### The relationship between sleep disturbances and sensory sensitivities

We examined the relationship between the total sleep disturbance scores and sensory sensitivity scores in each of the five sensory domains (Table 5). Significant negative correlations were apparent between the touch or oral sensitivity scores and total sleep disturbance scores of children with autism when computing Pearson’s or Spearman’s correlations. Control children exhibited significant negative correlations between the vestibular or touch sensitivity scores and total sleep disturbance scores when computing Pearson’s correlations, and similar trends were apparent when computing Spearman’s correlations. All other correlations were not statistically significant.

**Table 5:** Relationship between total sleep disturbance scores and total sensory scores in each of the sensory domains

	Autism (*n* = 69)		Control (*n* = 62)	
	Pearson	Spearman	Pearson	Spearman
Auditory	− 0.19	− 0.17	− 0.22	− 0.19
Visual	− 0.18	− 0.23	− 0.16	− 0.16
Vestibular	− 0.26	− 0.28	− 0.47*	− 0.33
Touch	− 0.54*	− 0.53*	− 0.42*	− 0.31
Oral	− 0.42*	− 0.41*	− 0.29	− 0.29

Pearson’s and Spearman’s correlations were computed for the autism (left) and control (right) groups. Asterisks indicate significant correlation coefficients after Bonferroni correction (*p* < 0.01)

**Fig. 4:**
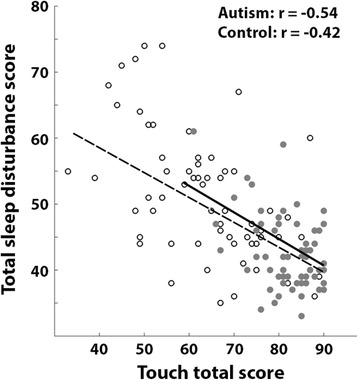
Relationship between total sleep disturbance scores and touch total scores. Scatter plot of autism (white) and control (gray) children. Pearson’s correlations are noted for each group. Each circle represents a single child. Dashed line indicates the linear fit for autism group. Solid line indicates the linear fit for the control group

Despite the strong correlations across sensory scores of the different modalities assessed by the Sensory Profile (Table 3), only *some* of the sensory scores were significantly correlated with the total sleep disturbance scores (Table 5). Furthermore, while significant negative correlations were apparent in the touch domain of both groups, scores of children with autism were distributed over a much wider range of values than control children as demonstrated in a scatter plot (Fig. 4). This means that the correlations in the autism group represented a tight relationship, which was apparent also in cases of severe sleep disturbances and sensory problems (i.e., correlation was robust throughout a larger range).

In additional analyses, we examined whether the total sleep disturbance scores were more strongly associated with low- or high-threshold items from the Sensory Profile (Table 6). Significant negative correlations were apparent between low item scores (i.e., hypersensitivity) in the touch and oral domains of children with autism and in the touch and vestibular domains of controls. Low item scores in all other sensory domains were not significantly correlated with sleep disturbance scores. Significant negative correlations were also apparent between high item scores (hyposensitivity) in the touch and oral domains of children with autism. High item scores in all other sensory domains of children with autism and all sensory domains in control children were not significantly correlated with sleep scores.

**Table 6:** Relationship between total sleep disturbance scores and hyper- or hyposensitivity scores in each of the sensory domains

		Autism (*n* = 69)		Control (*n* = 62)	
		Pearson	Spearman	Pearson	Spearman
Auditory	Hypersensitivity	− 0.13	− 0.13	− 0.30	− 0.24
	Hyposensitivity	− 0.21	− 0.14	− 0.05	− 0.12
Visual	Hypersensitivity	− 0.13	− 0.18	− 0.15	− 0.17
	Hyposensitivity	− 0.27	− 0.29	− 0.16	− 0.07
Vestibular	Hypersensitivity	− 0.08	− 0.14	− 0.46*	− 0.34
	Hyposensitivity	− 0.26	− 0.18	− 0.29	− 0.24
Touch	Hypersensitivity	− 0.50*	− 0.50*	− 0.42*	− 0.25
	Hyposensitivity	− 0.41*	− 0.36*	− 0.25	− 0.26
Oral	Hypersensitivity	− 0.35*	− 0.33*	− 0.31	− 0.32
	Hyposensitivity	−0.43*	− 0.44*	− 0.26	− 0.26

Pearson’s and Spearman’s correlations are presented for the autism (left) and control (right) groups. Asterisks indicate significant correlation coefficients after Bonferroni correction (*p* < 0.01)

### Explaining sleep disturbances with sensory sensitivity scores

In a final set of regression analyses, we quantified how much of the variance in total sleep disturbance scores could be explained by the total raw scores from each of the five sensory domains separately and also when including all of them together. Incorporating the total raw scores from all of the sensory domains into a single regression model yielded an adjusted *R*^2^ value of 0.29 in the autism group and 0.20 in the control group (Table 7).

**Table 7:** Variance explained by Sensory Profile scores

		Visual	Auditory	Vestibular	Touch	Oral	All
Raw scores (all items)							
Autism	*F* stat	2.61	2.22	4.67	28.3	14.2	6.64
	*p* value	0.11	0.14	0.03	*< 0.001*	*< 0.001*	*< 0.001*
	adj. *R*^2^	0.02	0.02	0.05	*0.29*	*0.16*	*0.29*
Control	*F* stat	2.93	1.81	15.8	12.8	5.67	3.96
	*p* value	0.45	0.18	*< 0.001*	*< 0.001*	0.1	0.015
	adj. *R*^2^	0.03	0.01	*0.2*	*0.16*	0.07	0.18
Low-threshold items only (hypersensitivity)							
Autism	*F* stat	1.22	1.1	0.4	22.2	9.2	5.38
	*p* value	0.27	0.3	0.53	*< 0.001*	*0.003*	*< 0.001*
	adj. *R*^2^	0.03	0	0	*0.24*	*0.11*	*0.24*
Control	*F* stat	5.87	1.4	16.2	12.8	6.46	4.12
	*p* value	0.5	0.24	*< 0.001*	*< 0.001*	0.05	0.015
	adj. *R*^2^	0.07	0	*0.2*	*0.16*	0.08	0.21
High-threshold items only (hyposensitivity)							
Autism	*F* stat	3.01	5.1	5.1	13.8	14.9	4.35
	*p* value	0.45	0.15	0.15	*< 0.001*	*< 0.001*	0.01
	adj. *R*^2^	0.03	0.06	0.06	*0.16*	*0.17*	0.2
Control	*F* stat	0.03	2.03	4.76	3.87	4.5	1.99
	*p* value	0.86	0.64	0.15	0.25	0.2	0.45
	adj. *R*^2^	0	0.02	0.06	0.04	0.05	0.07

Results of regression analyses using six different models: one model with a single predictor for each of the sensory modalities and a sixth model containing all five predictors together. This analysis was performed once with the total raw scores (i.e., sum of low- and high-threshold items) and again with the low- and high-threshold items separately. *F* stats, *p* values, and adjusted *R*^2^ are presented for each model. Italics indicate significant after Bonferroni correction (*p* < 0.01)

When performing the regression with a single predictor from each sensory modality separately, the touch scores stood out in their ability to explain sleep disturbance scores in children with autism (adjusted *R*^2^ = 0.29). This suggests that the touch scores could single-handedly explain as much of the variance in sleep scores as the integrated model, which contained all five predictors. In the control group, the vestibular and touch scores yielded adjusted *R*^2^ of 0.20 and 0.16 respectively, thereby demonstrating their ability to explain large portions of the variability in sleep scores in the control group.

We performed equivalent analyses while separating the scores from the low-threshold (hypersensitivity) and high-threshold (hyposensitivity) items. The full low-threshold regression model (containing five predictors/modalities) yielded adjusted *R*^2^ values of 0.24 and 0.21 in the autism and control groups respectively while the full high-threshold model yielded adjusted *R*^2^ values of 0.20 and 0.07. This demonstrates that low-threshold items that measure sensory hypersensitivity can explain sleep disturbance scores better than high-threshold items that measure hyposensitivity.

When examining low-threshold items in each sensory modality separately, the touch domain again stood out in its ability to explain sleep disturbance scores in children with autism (adjusted *R*^2^ = 0.24). This result demonstrates that low-threshold touch scores could single-handedly explain most of the variance that was explained by the full model with the total raw scores from all sensory modalities.

Adding age, gender, medication usage, and ADOS scores as additional predictors to the full models that contained all five predictors had negligible effects on the results. The adjusted *R*^2^ value improved from 0.29 to 0.30, demonstrating that these additional predictors explained only 0.2% of the variance in the sleep disturbance scores.

Taken together, these results suggest that total sleep disturbances in children with autism are most strongly associated with hypersensitivity towards touch. In contrast, sleep disturbances in control children are most strongly associated with vestibular hypersensitivity. Notably, scores in the visual and auditory sensory domains were remarkably weak in explaining sleep disturbances in both groups.

## Discussion

Our results reveal that sensory hyper- and hyposensitivity measures estimated using the complete Caregiver Sensory Profile can explain a considerable amount (29%) of the variance in sleep disturbance scores of children with autism (Tables 5, 6, and 7). In particular, hypersensitivity towards touch exhibited the strongest relationship with sleep disturbances and single-handedly explained 24% of the variability in sleep disturbance scores (Table 7). Similar, yet somewhat weaker relationships were also evident in the control group where touch and vestibular hypersensitivity scores explained up to 20% of the variance in sleep disturbance scores (Table 7). Interestingly, only hypersensitivity scores were significantly associated with sleep disturbance scores in the control group, but both hyper- and hyposensitivity scores were significantly associated with sleep disturbance scores in the autism group. This demonstrates the paradoxical overlap of sensory hyper- and hyposensitivity problems within the same children in the autism group (Fig. 2).

While one cannot infer causality from correlations, we speculate that hypersensitivity towards touch may interfere with sleep onset and sleep maintenance in children with autism, thereby generating severe sleep disturbances in children with touch sensitivities (Fig. 4). With this in mind, future studies examining sleep in autism may benefit from stratifying individuals with autism based on their sensitivity to touch as this measure may indicate the presence of a specific sleep disturbance mechanism.

### Specificity of sensory abnormalities associated with sleep disturbances

Are sleep disturbances associated with a general multi-modal sensory problem in autism, or with hyper- or hyposensitivity in particular sensory domains? Recent studies using the Short Sensory Profile have suggested that sleep disturbances are weakly associated with general sensory abnormalities, which explain 1–6% of the variance in sleep disturbance scores [21, 25]. The Short Sensory Profile, however, does not allow one to separate hypo- and hypersensitivity scores in individual sensory modalities.

Our in-depth assessment using the complete Sensory Profile reveals that sleep disturbances are not equally associated with sensitivities in all sensory modalities. In contrast to the moderate relationship between sleep disturbances and sensory problems in the touch domain, sensory problems in the visual and auditory domains were not associated with sleep problems in either the autism or the control groups (Tables 5, 6, and 7). Furthermore, sleep disturbances were more strongly associated with hypersensitivity towards touch than hyposensitivity (Tables 5, 6, and 7). Our results, therefore, clearly demonstrate that sleep disturbances are associated with sensory abnormalities in specific sensory modalities and cannot be generalized across all sensory domains. This highlights the need to use modality-specific measures of sensory sensitivity when studying autism.

### Sensory sensitivities, anxiety, and arousal

Sleep disturbances can be generated by a wide variety of interacting physiological and behavioral causes, which lead to hyper-arousal and insomnia [37]. Previous studies about sleep disturbances in autism have mostly highlighted the potential roles of anxiety [21, 25], poor sleep hygiene [20, 38], and a variety of physiological factors such as low endogenous levels of melatonin [39, 40]. These factors and others may create hyper-arousal and cortical over-reactivity to sensory stimuli, which in many cases can be ameliorated by behavioral and pharmacological interventions [41].

Our results suggest that hypersensitivity to touch may be an important factor in generating or exacerbating sleep disturbances in at least some children with autism. Further examination of the relationship between this specific sensory problem and the level of anxiety or arousal in individual children with autism is highly warranted. Furthermore, future studies could examine whether children with sleep disturbances and hypersensitivity to touch also exhibit excessive EEG responses to tactile stimuli, indicating cortical over-reactivity. Previous studies have revealed that EEG responses to auditory stimuli right before sleep onset and during different stages of sleep were abnormally strong in insomnia patients without autism [42–44]. Interestingly, it has been hypothesized that autism may be caused by the abnormal development of hyper-aroused and over-responsive neural circuits [45, 46].

### The relationship between sensory problems and sleep disturbances in typical development

Our findings are in line with several previous studies, which have also reported significant relationships between hypersensitivity on the Sensory Profile and sleep disturbances in infants [47] children [48] and adults [49] with typical development. Two of these studies examined sensitivity scores separately in each of the sensory modalities and reported that tactile hypersensitivity scores explained the largest amount of variability in sleep disturbance scores (~ 25%), while scores in other sensory domains, such as vision and audition, explained a considerably smaller portion of the variability [48, 49]. In our study, both vestibular hypersensitivity and hypersensitivity towards touch scores single-handedly explained a considerable amount of the variability in sleep disturbance scores of control children (20 and 16% respectively). Taken together, accumulating evidence suggests that hypersensitivity towards tactile and vestibular modalities is particularly useful for explaining sleep disturbances in children with typical development.

### Limitations of the study

This study has several limitations. First, our estimates of sleep quality and sensory sensitivities were based only on parental report. Previous research has shown that there is good agreement between parental report and objective techniques (i.e., actigraph and polysomnography) on some measures of sleep, but not all [50, 51]. For example, previous studies have shown that parental reports underestimate wakings during the night and overestimate sleep duration in typically developing children [52]. Second, control children in our study were not precisely matched to children with autism in terms of gender and we did not measure IQ in any of our participants. Third, while all of the children were diagnosed with autism by a child psychiatrist based on DSM V criteria, some of the children with autism did not complete a formal ADOS assessment. We addressed these issues by demonstrating that equivalent results were apparent in comparisons of specific subsets of the children (e.g., when including only male children or when including only the children with autism who had completed an ADOS assessment).

## Conclusions

The current study revealed that hypersensitivity to touch is likely to be an important factor in generating and/or exacerbating sleep disturbances in children with autism. This finding motivates further studies to examine this relationship with objective and more direct techniques such as psychometric and neuroimaging measures for tactile sensitivity as well as actigraph and polysomnography for sleep disturbances.

Characterizing the heterogeneity of autism and identifying individuals with shared symptoms and etiologies is a major goal of contemporary autism research [3, 53, 54]. With this in mind, we believe that stratifying children based on their hypersensitivity to touch may be important for elucidating the physiological mechanisms that generate sleep disturbances in autism and for determining the therapeutic effects of existing and new interventions and sensory aids.

## Abbreviations

ADOS: Autism Diagnostic Observation Schedule
ATN: Autism Treatment Network
CSHQ: Child Sleep Habits Questionnaire
